# A Review on the Synthesis and Applications of Mesostructured Transition Metal Phosphates

**DOI:** 10.3390/ma6010217

**Published:** 2013-01-15

**Authors:** Ronghe Lin, Yunjie Ding

**Affiliations:** 1 Dalian National Laboratory for Clean Energy, Dalian Institute of Chemical Physics, Chinese Academy of Sciences, Dalian 116023, China; E-Mail: linronghe@dicp.ac.cn; 2 State Key Laboratory of Catalysis, Dalian Institute of Chemical Physics, Chinese Academy of Sciences, 457 Zhongshan Road, Dalian 116023, China

**Keywords:** transition metal phosphate, mesostructure, synthesis method, application

## Abstract

Considerable efforts have been devoted to extending the range of the elemental composition of mesoporous materials since the pioneering work of the M41S family of ordered mesoporous silica by Mobil researchers. The synthesis of transition metal-containing mesostructured materials with large surface area and high porosity has drawn great attention for its potential applications in acid and redox catalysis, photocatalysis, proton conducting devices, environmental restoration and so on. Thus, various transition metals-containing mesoporous materials, including transition metal-substituted mesoporous silicates, mesostructured transition metal oxides and transition metal phosphates (TMP), have been documented in the literature. Among these, mesostructured TMP materials are less studied, but possess some unique features, partly because of the easy and facile functionalization of PO_4_ and/or P–OH groups, rendering them interesting functional materials. This review first introduced the general synthesis strategies for manufacturing mesostructured TMP materials, as well as advantages and disadvantages of the respective method; then, we surveyed the ongoing developments of fabrication and application of the TMP materials in three groups on the basis of their components and application fields. Future perspectives on existing problems related to the present synthesis routes and further modifying of the functional groups for the purpose of tailoring special physical-chemical properties to meet wide application requirements were also provided in the last part.

## 1. Introduction

As a kind of functioning material, transition metal phosphates (TMP) have been extensively studied in academy and widely used in industry. From the heterogeneous catalysis point of view, transition metal-containing mesoporous materials favorably combine the redox and/or acid-base catalytic properties of metal species with easy diffusion of reactant molecules. The most convincing example in this area would be vanadium phosphate, which is the only commercialized catalyst for the oxidation of butane to maleic anhydride. To date, several hundred papers have been devoted to their study, yet questions remain as to how they work, the optimal method of their preparation and, perhaps most significantly, the nature of the active vanadium phosphate phase [1]. Iron phosphates, another important oxidation catalysts, also showed high activity in a variety of oxidation reactions [2,3,4], among which the selective oxidation of methane to oxygenates is the most distinctive one [3]. Besides, they are extensively investigated as a kind of cathode electrode materials for the low cost, environmentally friendly and high theoretical specific capacity in lithium batteries [5]. Among the family of TMP, zirconium and titanium phosphates are the most studied members of solid acids [3,4]. Their electric behaviors, on the other hand, have been widely investigated. For example, Tian* et al.* found that an air electrode manufactured from mesoporous zirconium phosphate exhibited remarkable electrocatalytic activity for oxygen reduction reaction [6]. Ordered mesoporous zirconium phosphate films with a hexagonal structure showed a high proton conductivity of 0.02 S/cm parallel to the film surface at 80% RH and 298 K [7]. Titanium is also well known as a kind of good photocatalysis material. But, only the UV region of solar energy can be used for pure titania materials. Introducing foreign atoms into the framework of mesoporous titanium phosphates can lead to novel photocatalysts with an extended absorption region from UV to the visible region [8]. Due to the relatively high specific surface area and richness in surface hydroxyl group, mesoporous TMP materials have been frequently employed as adsorbents for radionuclide materials [9] and heavy metal ions [10]. Occasionally, mesoporous YPO_4_ materials, with or without the lanthanide metal ion dopants, showed interesting photoluminescence properties [11,12], which might be applied as drug delivery vehicles in biomedicine [13,14].

Precipitation of inorganic metal salts with a kind of phosphoric precipitating agent is commonly used in the preparation of bulky metal phosphates. The as-synthesized solids, however, usually have a low surface area, a low pore volume and irregular pore size distribution, which might greatly confine their applications, such as catalysis and adsorption. In this sense, advanced fabrication of mesoporous metal phosphates materials with greatly enhanced surface area and tunable pore structures might generate novel functional materials. The template method, presently, is probably the most important technique in the synthesis of mesostructured nanocomposites. Technically, the template method can be categorized into two groups,* i.e.*, soft-templating and hard-templating, based on various types of templates adopted. In a soft-templating route, ordered mesoporous materials can be self-assembled at the organic-inorganic interface with the assistance of surfactants or amphiphilic polymers [15], while ordered mesoporous materials, such as mesoporous silicates (SBA-15, MCM-41,* et al.*), carbon and aluminates, are used as the mother templates in a hard-templating route [16]. These templates can be completely removed by high-temperature calcination, solvent extraction or oxidation, but residuals might remain in some cases [17,18,19]. Based on the templating route, a variety of mesoporous materials have been successfully synthesized, and the mesoporous framework can be expanded significantly from silicate to non-silicate, including carbon [20], metal/alloy [21,22], metal oxide/metal sulfide/metal phosphate [23,24,25] and nitride [26]. Among all these mesostructured composites, well-written review articles are available for carbon [27], metal [28] and metal oxide [29], not to mention tremendous reviews on ordered mesoporous silicates [30,31,32,33].

In this review, we attempt to summarize the preparation methods and applications of mesoporous TMP materials. Firstly, general synthesis strategies for mesoporous materials are introduced, which are also applicable to the manufacturing of TMP materials. While the templating methods are emphasized here, recent developments in template-free strategies are also briefly discussed. Secondly, the fabrication of mesoscaled TMP materials is grouped into three categories for discussion. Based on the application fields, zirconium and titanium phosphates with versatile functions in various fields are classified as the first group. Iron, vanadium, and nickel phosphates belong to the second group, since these components are basically employed in catalysis. The other TMP components, including chromium, niobium, zinc, tantalum and yttrium phosphates, are put into the third group, for the respective materials are relatively less studied and only used in some special applications. Lastly, perspectives are provided on urgent problems associated with the template method, further exploring novel synthetic strategies and tailoring surface properties by functionalization of mesoporous transition metal phosphates.

## 2. General Synthesis Strategies

### 2.1. Structure-Directing Synthesis Routes

#### 2.1.1. Soft-Templating Route

**Figure 1:**
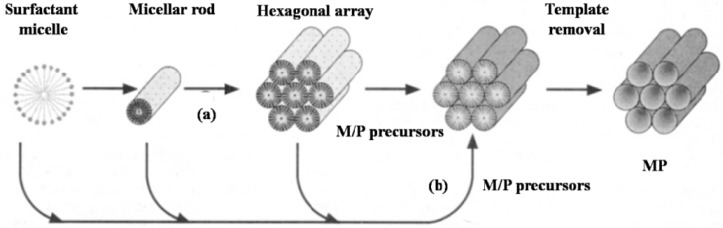
Procedures for soft-templating synthesis strategy: (**a**) liquid-crystal templating mechanism and (**b**) cooperative formation mechanism. M, P and MP represent metal, phosphorus and metal phosphates, respectively (Reprinted with permission from [15]. Copyright 1992 American Chemical Society).

The generally adopted pathway for creating ordered mesoscaled materials is organic-inorganic assembly combined with sol-gel and/or hydrothermal processes by using various surfactants as structure-directing agents. As far as synthesis is concerned, the synthetic components of mesoporous materials have been extensively extended to alumina, pure metals, transition metal oxides and even various metal phosphates and/or phosphonates [20,21,23] since the first report of a family of highly ordered mesostructured silicates M41S materials by Mobil scientists [34]. The synthesis of all the above mentioned mesoporous materials is believed to be based on a similar templating mechanism, liquid crystal templating (LCT), in which various different surfactant liquid crystal structures serve as organic templates. Two possible pathways were proposed for the LCT mechanism, as shown in Figure 1. The difference lies in whether the liquid crystal phase is intact before the addition of inorganic species or the ordering of the subsequent surfactant micelles is the result of precursors addition. Even so, the resultant inorganic components can mimic known crystal phases after the removal of templates in either case [15].

A wide range of mesostructured materials have been successfully prepared with the aid of various surfactants, and these synthesis routes can be roughly classified into three categories based on the surfactant added. Ionic surfactants can be used as a template for self-assembly with the charge reversed ionic inorganic precursor to form a definite structure by coulomb interactions (S^+^I^−^ or S^−^I^+^). Another route involves counterion (X^−^ or M^+^), where the surfactant and the inorganic species have the same charge (S^+^X^−^I^+^, S^−^M^+^I^−^). A neutral templating route uses non-ionic surfactant (S^0^), and the self-assembly in this case is based on hydrogen bonding between a neutral inorganic precursor and a neutral template agent [35,36]. The assembly process is very complicated, and there are several main factors that could influence the structural properties of resultant materials when using the soft-templating method. As has been stated, the as-synthesized mesoporous materials will have the intact micellar structures, suggested by the liquid crystal mechanism, so, the nature of selected surfactants and any factors that can influence the formation of surfactant micelle would have a great impact on the final structural properties. Taking MCM-41 for example, when quaternary ammonium surfactants (C*_n_*H_2*n*+1_(CH_3_)_3_)N^+^ with different alkyl chain lengths were adopted, the resultant MCM-41 materials exhibited different locations of *d*_100_ X-ray diffraction lines and distinctively different pore sizes; long surfactant chain length led to higher *d*_100_ spacing and larger pore size [15]. Besides, the morphologies of MCM-41 from hexagonal to cubic and even other less well defined phases could also be tuned simply by changing the surfactant/Si ratios, for the variation both in the ionic strength and the surfactant concentration can induce changes in liquid crystal phase [15].

Great success has been achieved in the preparation of mesoporous silicate materials, but the direct grafting of this soft-templating method from silicate to non-silica components, such as metal oxides and phosphates, presents great difficulties. This is mainly due to the rather rapid hydrolysis of inorganic metal precursors in acidic solutions, making it rather difficult to introduce mesostructures into liquid crystal template. In some attempts for the synthesis of ordered mesoporous metal phosphates, the incorporation of phosphonate into the resultant materials was found to be less than 50% and greatly restricted on the pore surfaces [37]. Considering the fast hydrolysis of metal precursors in acidic aqueous solutions, Yang* et al.* successfully prepared various mesostructured metal oxides in non-aqueous solutions via an evaporation-induced self-assembly (EISA) process, with amphiphilic poly(alkylene oxide) block copolymers as structure-directing agents [38]. Later, Tian* et al.* described a new perspective in which the self-adjusted inorganic–inorganic (II) interplay between two or more inorganic precursors is guided by acid–base chemistry considerations [39]. They further demonstrated the versatility and validity of “acid-base pair” guide through the successful synthesis of a wide variety of highly ordered, large-pore, homogeneous, stable and multicomponent mesostructured minerals, including metal phosphates and metal borates, as well as various metal oxides and mixed metal oxides. Undoubtedly, the “acid-base pair” synthesis route provides a good deal of insights into the fundamental processes involved in the EISA process and sol-gel process, thus promoting the great developments of mesoporous non-silicates materials. Compared with the silicates precursor, the precursor of inorganic metal salts is largely restricted in number. Due to the difficulty in controlling the hydrolysis and polymerization processes of inorganic metal precursors, the final yielded materials with high purity are hardly available. The resultant metal-containing materials generally possess less ordered mesostructures and poor thermal stability because of the decomposition of templates at relatively low temperatures. For these reasons, the fabrication of metal-containing mesostructured materials with good regularity and high thermal stability is still a big challenge.

#### 2.1.2. Hard-Templating Route

**Figure 2:**
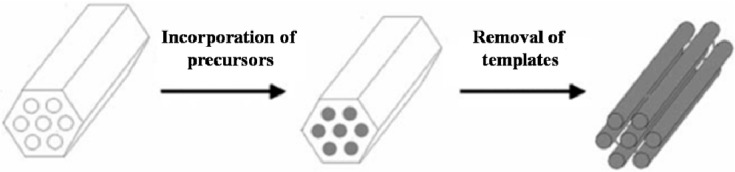
Representative scheme for hard-templating synthesis strategy (reprinted with permission from [45]. Copyright 2005 Royal Society of Chemistry).

Since many mesoporous materials cannot be prepared by the soft-templating route, researchers have developed various novel mesoscaled materials by the hard-templating route, also known as nanocasting technology (shown in Figure 2). This relatively late emerging synthetic method is initially proposed by Martin, who first prepared porous alumina anodic membranes by using etching techniques and then used them as the hard template to synthesize nanoscaled polymers, metals, semiconductors and other materials via eletrodeposition or chemical vapor deposition methods [40]. One straightforward advantage of the nanocasting technology is that the developed materials can completely replicate the topologies of the templates after the removal of the templates. The initially employed alumina template by Martin was not so regular, with a relatively broad pore size distribution. Considering the stability and regularity in structure, hard template materials have been extended to mesoporous silicate, alumina/aluminum composite oxide and carbon [41,42]. By far, mesoporous silicate materials with highly ordered structures and uniform pore size, such as SBA-15 [41], KIT-6 [43] and FUDU-5 [44], are the most studied and ideal ones.

The hard-templating route can be viewed as an extension of the templating concept in the preparation of zeolites and mesoporous materials. Highly ordered mesoporous silicates are employed as hard templates instead of organic amines or surfactants used in zeolites preparation. The precursors can be introduced into the inner pores or channels of mesoporous silicates by sorption, ion exchange, phase transition, complex or covalent grafting. The precursors are thermally decomposed after heat treatment, while the resulting nanoparticles gradually grow larger by interconnection to each other combined with a crystallization process. As a consequence, the byproducts generally leave the pores or channels in the gas phase. During the thermal decomposition process, the nanocrystals may interlink with each other and form continuous frameworks, given that the loading rate of the precursors is high enough. After the templates are removed by dissolution or calcination, the mesostructures are fully copied by the emerging nanocrystals, and replica materials are obtained. The most important point in the success of hard-templating route is related to the impregnation of precursors. During this process, the precursors tend to be absorbed on the external surface of the templates rather than the mesopore channels. Different impregnation methods have been proposed, such as evaporation [46], solid-state grinding [47] and the so-called “two solvents” impregnation methods [48]. The essence of all the developed methods is to increase the loading rate of the precursors in the mesopore channels of the templates. Scott and coworkers [49] indicated that capillary effect is the driving force for promoting the movement of the precursors into the mesopores. When a silicate template is involved, the capillary function might be suppressed because of the weak interaction between the silicate walls and the precursors. In such cases, the precursors would largely remain outside the mesopores and/or stick inside the mesopore channels and/or block the channels. Therefore, the fabrication of ordered mesostructured materials can frequently fail after removing the silicate templates.

Nanocasting using three-dimension ordered mesoporous silicate as a hard template is one of the most important strategies for the synthesis of highly ordered non-siliceous mesoporous materials, such as metal [50], metal oxides [51], carbon [52], carbide [53] and nitride [54]. Unfortunately, up to now, demonstration of successful fabrication of ordered mesoporous metal phosphates is quite rare. As we know, the established synthetic methods for metal phosphates are substantially based on the precipitation reaction between soluble metal salt and phosphate. However, the traditional precipitation is not suitable for the nanocasting route. Since the precipitation of metal phosphates is a much faster process as compared with the loading of both metal and phosphorous precursors into the mesopores of templates, the precipitation might probably occur outside the mesopores and even usually block the pore channel if the precipitation is too fast inside the mesopore channels, resulting in the formation of disordered nanoparticles. By using the Y(NO_3_)_3_/H_3_PO_4_/HNO_3_ system as a guest unit and KIT-6 silica as a hard template host, Luo *et al.* [12] successfully synthesized cubic ordered mesoporous YPO_4_ material. This report puts forward some new ideas about the preparation of mesoporous metal phosphates with a guest unit containing both metal and phosphate ions and a controlled precipitation process.

### 2.2. Template-Free Synthesis Routes

Template-directing is now the predominant preparative route in the synthesis of ordered mesoporous materials, including mesostructured metal phosphates. Nevertheless, surfactants or templates are frequently used in the reaction process, which will require expensive reagents and involve complex procedures. Additionally, the complete removal of surfactants and templates to meet some specific application is problematic. So it is still a big challenge in exploring a facile, cost-effective, high-yield and green method to synthesize mesoporous metal phosphates without surfactants or templates. Several novel template-free synthesis methods have been proposed and proved to be effective in the manufacture of mesostructured metal phosphates. Ma and coworkers developed a new method to prepare metal phosphates consisting of wormhole-like mesopores and mesostructured cellular foam (MCF) by using microemulsion [8]. They further demonstrated that the formation of phosphate-related hybrid materials in a microemulsion system can result in mesoporous materials with different morphologies and that the organic additive and pH value of the reaction solution have a great impact on the morphology and structural properties of the resulting mesoporous metal phosphates. More recently, Qian* et al.* reported the synthesis of amorphous mesoporous FePO_4_ nanoparticles by a new cost-effective electrochemical method and employed the resultant nanoparticles to prepare LiFePO_4_/C nanocrystals as cathode materials [55]. Topological transformation can lead to mesoporous materials when the precursors employed possess some layered or porous characteristics. For example, layered metal organophosphonates, with aryl- and alkylphosphonate molecules coordinated to metal oxide layers, can be used to synthesize mesoporous metal phosphates. In these structures, aryl functional groups are suited in the interlamellar spaces, but the small spaces between aryl pillars prevent the formation of pores [56]. By modifying the aryl pillars with smaller functional groups like phosphoric or phosphorous acids, the “close-pillar deposition” can be overcome and mesoporous hybrid materials with enhanced specific surface area can be generated [57,58]. Using a special layered vanadyl *n*-butylphosphate as the precursor, Kamiya *et al.* synthesized highly porous vanadium phosphates of high specific surface area by thermal treatment of layered vanadyl *n*-butylphosphate in N_2_ atmosphere [59]. The authors suggested that the mesopores, with a width of 4.4 nm, were probably formed from the fractures of microcrystallites along layers of vanadyl *n*-butylphosphate. Thermal decomposing an inorgano-organic precursor of Zr[(O_3_P–C_6_H_4_–PO_3_)_0.54_(O_3_P–C_6_H_4_–PO_3_H_2_)_0.13_(O_3_PH)_0.79_] led to mesoporous zirconium phosphate-pyrophosphate exhibiting similar morphologies and pore structure as those in the precursor [60]. By post-treating a zirconium oxide mesophase with phosphoric acid, porous zirconium phosphate materials were prepared with the same ordered hexagonal pore system in the oxide [61]. There is no doubt that all these novel preparation methods have great inspiration for the fabrication of mesoporous TMP materials, although more fundamental work should be taken so as to shed light on deep understanding of the template-free synthesis of mesoporous materials in a more general way.

## 3. Synthesis and Application of Mesostructured Transition Metal Phosphates

### 3.1. Zirconium Phosphates and Titanium Phosphates

#### 3.1.1. Zirconium Phosphates (ZrP)

**Figure 3:**
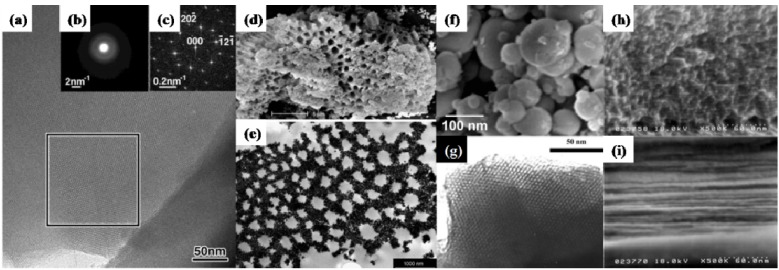
Transmission electron microscope (TEM)/scanning electron microscope (SEM) images of mesoporous ZrP materials. (**a–c**) Porous mesostructured zirconium oxophosphate with cubic symmetry synthesized by C18BDAC surfactant-assisted precipitation (reprinted with permission from [63]. Copyright 2002 American Chemical Society). (**a**) The High-resolution transmission electron microscopy (HRTEM) image taken along the [111] zone axis; (**b**) The electron diffraction pattern; (**c**) The Fourier diffractogram obtained from the HRTEM image in the labeled rectangular area; (**d**,**e**) Hierarchically nanostructured porous ZrP synthesized by Brij 56 (C_16_(EO)_10_ assisted self-assembly (reprinted with permission from [68]. Copyright 2005 Elsevier); (**f**) Mesoporous ZrP with spherical particles morphology, prepared by sequential precipitation-hydrothermal procedure in basic medium (reprinted with permission from [72]. Copyright 2006 Elsevier); (**g**) Hexagonally packed porous ZrP derived from anion exchange between zirconium oxide mesophase and phosphoric acid (reprinted with permission from [61]. Copyright 2005 American Chemical Society); (**h**,**i**) Ordered mesoporous ZrP films obtained by spin coating and vapor treatments (reprinted with permission from [7]. Copyright 2006 American Chemical Society).

Zirconium phosphates are one of the most studied materials in the family of transition metal phosphates. Various methods have been proposed for the fabrication of mesoporous ZrP, with the synthesis conditions, structures and morphologies and applications of resulting materials summarized in Figure 3 and Table 1. Ciesla and coworkers [62] synthesized a porous zirconium oxo phosphate with a surfactant-assisted synthesis method. Zr(SO_4_)_2_·4H_2_O and hexadecyltrimethylammonium bromide were employed as the zirconium and template, respectively. A colorless solid product can be obtained through a simple precipitation process. The zirconia compound with surfactant filled might have a 2D hexagonal structure similar to MCM-41, as confirmed by the small-angel X-ray diffraction. After annealing at 773 K, the low angle reflections disappeared, suggesting the collapse of the ordered pore structure. The scientists then enhanced the thermal stability of the uncalcined sample by treating with phosphoric acid. As a result, porous zirconium phosphate with a less ordered structure and a surface area of 400 m^2^/g was finally synthesized after heat treatment [62]. It is worthy to note that the thermal enhancement of mesoporous metal phosphates with phosphoric acid was afterwards widely adopted by other research groups [63,64]. Mesoporous ZrP with high surface area and narrow size distribution was later synthesized by Jimenez-Jimenez* et al.* in a similar method, using an aqueous solution of cetyltrimethylammonium (CTMA) bromide and orthophosphoric acid and zirconium *n*-propoxide in a sol-gel approach [65,66]. The later method differs from the former in that phosphoric acid was added directly at the stage of the formation of the inorganic matrix. Besides, another template removal procedure besides calcinations was proposed by acid-ethanol extraction [65].

The templating synthesis of mesoscaled ZrP is presently the most popular route. Various precursors of zirconium have been explored, including Zr(SO_4_)_2_ [63], ZrOCl_2_.8H_2_O [67], Zr(OC_3_H_7_)_4_ [68] and Zr(OC_4_H_9_)_4_ [69]. Correspondingly, surfactants of cationic, neutral and anionic types were employed, depending on the adopted zirconium precursor and preparative route; typical examples are listed as follows: C18BDAC [63], cetyltrimethylammonium chloride (CTAC) [67], hexadecylamine (HDA) [67], sodium dodecyl sulfate (SDS) [67], Brij 56 (C_16_(EO)_10_ [68], pluronic-F127 [69], sodiumbis(2-ethylhexyl)sulfosuccinate (AOT) [70] and octadecyltrimethyl ammonium bromide (OCTBr) [71]. The inorganic precipitation agents can be H_3_PO_4_ [67,68], NH_4_H_2_PO_4_ and (NH_4_)_2_HPO_4_ [28] and PCl_3_ [69], leaving H_3_PO_4_ the most favorably chosen one. It should be noted that the above-mentioned surfactant-assisted precipitation methods generally proceed in an acidic solution (phosphoric acid or hydrochloric acid commonly adopted). In a recent report, Tarafdar *et al.* [72] synthesized mesoporous ZrP with spherical particles morphology (Figure 3f) and excellent acidic properties using a zirconium carbonate complex, (NH_4_)_2_HPO_4_ and tetradecyltrimethylammonium bromide (TTBr) in a basic medium through a sequential precipitation-hydrothermal treating process. The mesostructured ZrP had a specific surface area of 299 m^2^/g with narrow pore size distribution and was used successfully as an acid catalyst in ethyl acetate hydrolysis reaction in liquid phase. It is the first report that mesoporous ZrP material was fabricated in a basic medium. Additionally, mesoporous ZrP can also be derived from yeast biotemplate of glucose [6]. The biotemplated mesoporous ZrP showed a disordered wormhole-like morphology, with a specific surface area of 217 m^2^/g and a narrow pore size distribution centered at 2.7 nm. Amide carboxyl groups of yeast were found to play an important role in the chemical interaction between protein molecules and ZrP nanoparticles. An air electrode fabricated from mesoporous ZrP exhibits remarkable electrocatalytic activity for oxygen reduction reaction, compared to that of the commercially employed electrolytic manganese dioxide air electrode [6].

Ordered mesoporous zirconium phosphate films (Figure 3h,i) could also be prepared on a silicon substrate by spin coating. Post-vapor treatments with phosphoric acid and ammonia on the spin-on film were found to effectively enhance the thermal stability of the ordered mesostructure [7]. The calcined ZrP film has a hexagonal structure with straight channels parallel to the film, exhibiting a high proton conductivity of 0.02 S/cm parallel to the film surface at 80% RH and 298 K and showing potential in the application of proton conducting devices. Wu* et al.* [61] prepared porous ZrP with an ordered hexagonal pore system (Figure 3g) by post-treating surfactant-assisted zirconium oxide mesophase with phosphoric acid. ZrP with P/Zr atomic ratios over 1.0 were thermally stable up to 873 K and showed a surface area of 456~547 m^2^/g. The formation mechanism of the hexagonal ZrP was clarified to be essentially an anion exchange of HPO_4_^2−^ ions for SO_4_^2−^ ions, which mediated the cationic zirconium species and the cationic surfactant headgroups within the as-synthesized zirconium oxide mesophase through electrostatic interactions, together with a reaction between the phosphoric acid and the Zr–OH groups [61]. By thermal decomposing a special inorgano-organic precursor of mesoporous zirconium phosphate pyrophosphate, mesoporous zirconium phosphate-pyrophosphate was derived with a large amount of thermally stable acid groups on the pore surface [60]. These acid groups remain, even up to 973~1073 K, showing potential uses as an acid catalyst in high temperature reactions. It is interesting to note that both the precursor and the thermal resultant materials have the similar morphologies and pore structures. This might suggest the topotactic transformation from precursor to the resultant materials. Though it seems a straightforward approach, scaled-up production is unrealistic due to less variety and the high cost of the inorgano-organic precursors.

**Table 1:** Summary on the synthesis, structural property and application of mesostructured ZrP.

Materials	Synthesis conditions	Physical properties	Applications	Reference
ZrP	Yeast as biotemplate, assembly, ambient conditions	Wormhole-like mesoporous structure, S_BET_* ca.* 217 m^2^/g, narrow pore size of 2.7 nm	Electrode for oxygen reduction reaction	[6]
	Spin coating, P123, zirconium isopropoxide, triethyl phosphate	Ordered mesoporous films, hexagonal structure	Proton conducting devices	[7]
	Coassembly, pluronic-F127, zirconium butoxide, phosphorous trichloride	Randomly ordered mesostructures, S_BET_ 84 m^2^/g, average pore size of 17 nm	Nafion-zirconium phosphate composite membranes	[39,69]
	Thermal decomposing a mesoporous zirconium phosphite diphosphonate	Globular particles of 10~20 nm diameter, S_BET_ 215 m^2^/g, average pore size *ca.* 4.0 nm	Potential uses as an acid catalyst at high temperature	[60]
	Post-treating surfactant-assisted zirconium oxide mesophase with phosphoric acid	Ordered hexagonal pore, S_BET_ 456~547 m^2^/g, pore size of 1.30~1.66 nm	Untested	[61]
	Precipitation of zirconium sulfate, followed by hydrothermal treating	Ordered mesostructures, S_BET_ 230~390 m^2^/g	Untested	[62]
	Supramolecular, self-assembly (C18BDAC), aging at 363 K, 3 day	Cubic *Ia*3*d* structure	Potential solid acid catalyst (Brønsted and Lewis acid sites)	[63]
	Precipitation of zirconium sulfate with gemini cationic surfactants, followed by hydrothermal treating	Highly ordered mesostructures	Untested	[64]
	Sol-gel, CTMA template, aging at room temperature 2~3 day	Less ordered mesoporous structure, S_BET_ 250~320 m^2^/g, average pore size 2.5~2.7 nm	Proton conducting devices, potential solid acid catalyst	[65,66]
	Surfactant-assisted precipitation (CTAC, HDA, SDS), aging 24 h	Porous, S_BET_ 400~500 m^2^/g	Untested	[67]
	Precipitation of (Zr(OC_3_H_7_)_4_ with Brij 56, followed by hydrothermal treating	Hierarchical structure with supermicroporous walls, uniform diameters ranging from 300 to 800 nm	Potential applications in catalysis	[68]
	“Surfactant-assisted” approach (AOT)	Porous, S_BET_ 83 m^2^/g, pore size of 2~30 nm	Support for protein adsorption (myoglobin)	[70]
	Precipitation of a zirconium carbonate complex, pH 8.0	Spherical, S_BET_ 299 m^2^/g, narrow pore size of 3.91 nm	Ethyl acetate hydrolysis	[72]
	Evaporation-induced self-assembly, F127, strongly acidic conditions	Wormhole-like disordered mesostructure, S_BET_ 260~312 m^2^/g, narrow pore size of 4.5~5.5 nm	Conversion of the long chain fatty acids to their respective methyl esters	[73]

#### 3.1.2. Titanium Phosphates (TiP)

**Figure 4:**
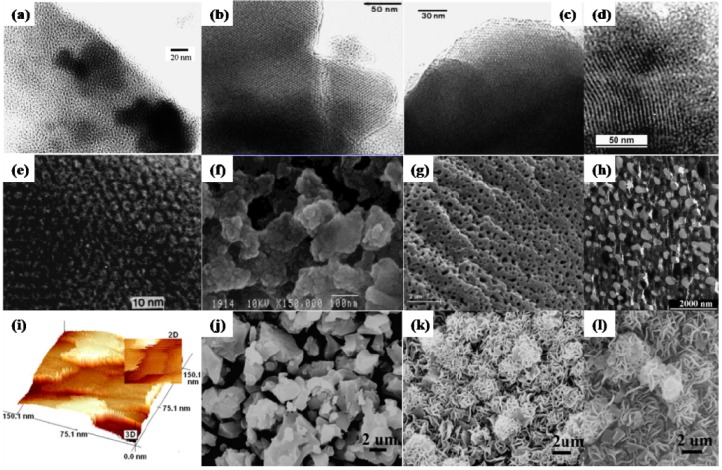
TEM/SEM/AFM (Atomic force microscope) images of mesoporous TiP materials. (**a**) Mesoporous titanium oxo phosphate with a disordered structure synthesized by using a low-cost industrial polyethylenoxide named Dodecanol +5 EO (BASF ) as a non-ionic surfactant (reprinted with permission from [76]. Copyright 1999 Elsevier); hexagonal structured ZrP materials fabricated with the assistance of surfactants (**b**) C_18_TAB and (**c**) C_16_TAB (reprinted with permission from [77]. Copyright 2000 Elsevier); (**d**) Mesoporous TiP prepared by reaction between phosphoric acid solution and titanium chloride in the presence of trimethylammonium surfactants (reprinted with permission from [74]. Copyright 2000 Royal Society of Chemistry); (**e**) TCM-7 and (**f**) TCM-8 with poorly ordered two-dimensional hexagonal mesophase, synthesized by surfactant-assisted assembly (reprinted with permission from [37]. Copyright 2001 American Chemical Society); (**g**,**h**) Hierarchical TiP materials with multiple porosities of different lengths (meso-macroporous and meso-macro-macroporous) derived from a self-formation process [(Ti(OC_3_H_7_)_4_-Brij 56-H_3_PO_4_] (reprinted with permission from [79]. Copyright 2006 American Chemical Society); (**i**) Mesoporous TiP with unique lamellar structures and mesopores on the surfaces fabricated by a yeast cell-assisted bio-templating route (reprinted with permission from [80]. Copyright 2011 Springer). Mesostructured TiP materials with various morphologies, prepared by a C_16_TAB-assisted hydrothermal process at different temperatures (**j**) room temperature; (**k**) 348 K and (**l**) 373 K (reprinted with permission from [78]. Copyright 2007 Elsevier).

A family of novel mesoporous TiP materials has been prepared in the presence of a structure-directing surfactant, mainly by sol-gel synthesis, in which interactions are exerted between the inorganic species and individual organic molecules of the surfactant in a cooperative nucleation process. The synthesis conditions, structures and morphologies and the applications of the resulting TiP materials are summarized in Figure 4 and Table 2.

Jones* et al.* reported a mesoporous form of TiP (Figure 4d), prepared by reaction between phosphoric acid solution and either titanium propoxide or titanium chloride in the presence of trimethylammonium surfactants [74]. Although not well ordered in the long range, TEM images showed local ordering of channel structures, which are identified as disclination effects well known in liquid crystal systems [75]. Interestingly, these mesostructured materials showed a very high surface area of around 740 m^2^/g, and an extraordinarily high surface density of acid sites (900 mmol/g ammonia adsorbed from the gas phase at 353 K) because of the richness in hydrogen phosphate groups (HPO_4_^2−^ and H_2_PO_4_^−^) [74]. Investigation of the properties of mesoporous TiP in fine chemical synthesis and acid catalysis should be of interest.

Mesoporous titanium phosphates of new mesoporous cationic framework topologies, namely, TCM-7 and -8 (Toyota Composite Materials, numbers 7 and 8), were further synthesized by the scientists from Toyota company, using both cationic and anionic surfactants as structure-directing agents (see Figure 4e,f) [37]. They claimed that TiCl_4_ is a suitable Ti source for anionic surfactants, while Ti-alkoxide is the most suitable for the synthesis of these TiP in the presence of cationic surfactants. Quite small and uniform cubic to spherical crystals of 20~60 nm in size forming large spherical aggregates of 0.1~0.2 µm were observed in these composites, and poorly ordered two-dimensional hexagonal (*p*6*mm*) mesophase were confirmed by TEM. Mesoporous TCM-7 and -8 show anion exchange capacity due to the existence of defective P–OH groups. Besides, most of the Ti in these materials is confirmed to be tetrahedrally coordinated [37], showing potential as liquid-phase oxidation catalysts.

A new mesoporous titanium oxo phosphate (Figure 4a) was synthesized by using a low-cost industrial polyethylenoxide named Dodecanol +5 EO (BASF ) as a non-ionic surfactant [76]. The formation of the mesoporous structure is dominated by hydrogen bonding between self-assembled surfactant micelles and inorganic precursor of Ti(OMe)_4_. The derived titania–surfactant composite was thermally enhanced with phosphoric acid, followed by a typical template removal method via calcination. N_2_-adsorption revealed the existence of mesoporosity in these titanium oxo phosphates with a surface area of 350 m^2^/g and pore sizes approximate to 4.5 nm. The materials are thermally stable up to 823 K, though there is no well defined hexagonal structure. In order to create more regular pore structures, the same researcher group developed a novel (Ti(OPr*^i^*)_4_-sulfuric acid-alkyltrimethylammonium bromide (C*_x_*TAB, *x* = 16; 18 or 20) system [77]. Depending on the chain length of the surfactant and the calcination temperatures, hexagonally or lamellarly packed porous titanium oxo-phosphate (Figure 4b,c) can be tuned. While C_16_ or C_18_ surfactants resulted in hexagonal structures, C_20_ surfactant led to a lamellar structure. Using a Ti(OPr_4_)-C_16_TAB-H_2_SO_4_-H_3_PO_4_ system, Wang and coworkers [78] observed the morphological transformations of mesostructured TiP from hexagonal to lamellar structures (Figure 4j–l) simply by increasing the hydrothermal treating temperature from room temperature to 373 K. No further morphology changes were observed for the crystalline materials treated at higher temperatures. Characterization results revealed that the change of Ti coordination sites played an important role in modifying the charge balance and conformation of surfactant groups, which in turn result in phase transformation [78].

Hierarchical TiP materials with multiple porosities of different lengths (meso-macroporous and meso-macro-macroporous) (Figure 4g,h) were synthesized by a self-formation process [(Ti(OC_3_H_7_)_4_-Brij 56-H_3_PO_4_] [79]. The porous hierarchy could be further tuned by using the poly(ethylene oxide) surfactant technique [79]. Based on the principles of microbial fermentation, cytoarchitectonics and biomineralization, He and coworkers developed a novel synthetic method by which the multilayered nanostructure of yeast cell tissues can be copied by phosphates [80]. Mesoporous TiP with unique lamellar structures and mesopores on the surfaces can be synthesized (Figure 4i). Other hierarchically mesoporous phosphate nanocomposites, such as Ca-, Zn- and Mg-phosphates, can also be prepared using similar method [80]. These synthesized nanocomposites might have potential applications in the biomedical field, such as drug-release carriers, immobilized enzyme and gene and/or cell carriers.

Other templating synthesis systems of mesoporous TiP materials are proposed, including [Ti(OC_3_H_7_)_4_-CH_3_(CH_2_)*_n_*NH_2_-ethanol-H_3_PO_4_] [81], [Ti(*i*-PrO)_4_-P123-H_3_PO_4_-NH_4_OH] [9], [TiCl_4_-Brij 56-ethanol-EDTMPS] [82] (EDTMPS: Sodium salt of ethylene diamine tetra(methylene phosphonic acid)),* etc.* Due to the commonly existent acidic sites and ion-exchange capabilities, these mesoporous TiP materials were applied in a variety of fields such as solid-acid and photocatalysts [82] and adsorbents of heavy metal ions [82] and radioactive elements [9].

**Table 2:** Summary on the synthesis, structural property and application of mesostructured TiP.

Materials	Synthesis conditions	Physical properties	Applications	Reference
TiP	Template-directing assembly (P123, Tergitol 15-S-9, CTAC), pH 4	S_BET_ 107~340 m^2^/g, pore size of 4.0~4.5 nm	Radionuclide sorbent materials Np(V)	[9]
	Template-directing assembly and hydrothermal, SDS, DBSA, ODTMABr/Cl	Uniform hexagonal mesopore, S_BET_ 407~701 m^2^/g, average pore size of 2.18~3.13 nm	Ion-exchange	[37]
	Precipitation of titanium propoxide or titanium chloride with H_3_PO_4_ in the presence of trimethylammonium	S_BET_ 207~740 m^2^/g, average pore size of 2.3~5.1 nm	Potential acid catalyst	[74]
	Non-ionic template route, [Dodecanol +5 EO]	Disordered hexagonal pore structure, S_BET_ 350 m^2^/g, average pore size *ca.* 4.5 nm	Untested	[76]
	Precipitation of titanium isopropoxide with surfactant CTAB	Hexagonally packed porous structure or lamellar structure,	Untested	[77,78]
	Self-formation process, hydrothermal 353 K 24 h, with/without Brij 56	Disordered framework with wormhole-like channels, S_BET_ 165~312 m^2^/g	Potential optical material and acid catalyst	[79]
	Yeast cells induced self-assembly	Lamellas with mesopores, pore size 3~12 nm	Untested	[80]
	Neutral templating route, hydrothermal aging at 363 K 48 h, long-chain *n*-alkyl amine	Wormlike mesopore, S_BET_ 359~497 m^2^/g, average pore size of 1.7~3.3 nm	Liquid-phase partial oxidation of cyclohexene with H_2_O_2_	[81]
	Hydrothermal combined with evaporation-induced self-assembly, Brij 56	Ordered hexagonal pore structure, S_BET_ 230~1021 m^2^/g, average pore size of 2.6~3.4 nm	Photocatalysts for organic dye degradation, adsorbents for heavy metal ions	[82]

### 3.2. Iron, Vanadium and Nickel Phosphates

**Figure 5:**
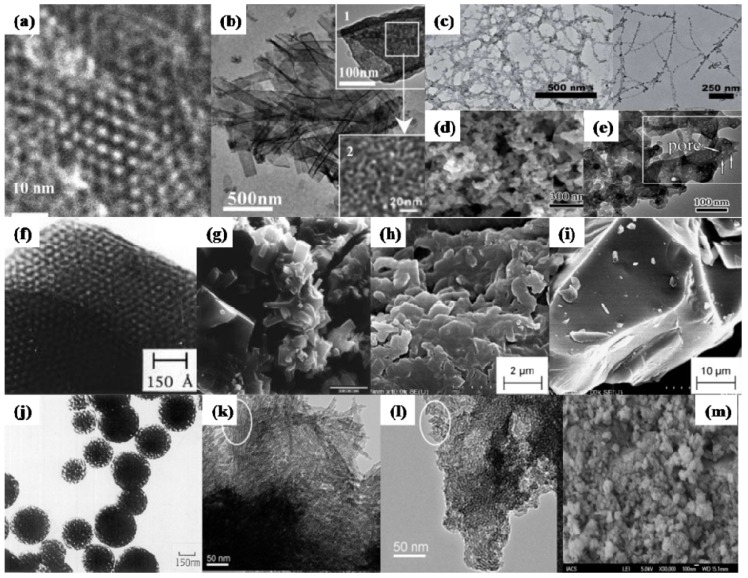
TEM/SEM images of mesoporous iron (**a–e**), vanadium (**f–i**) and nickel phosphates (**j–m**) synthesized by various methods. (**a**) Ordered mesostructured FeP prepared with the HF assembly method (reprinted with permission from [97]. Copyright 2007 American Chemical Society); (**b**) Nanotubular and mesoporous FeP synthesized in a modified fluoride route with the aid of sodium dodecyl sulfate (SDS) (reprinted with permission from [86]. Copyright 2007 American Chemical Society); (**c**) Amorphous FeP nanowires derived from a M13 virus-based bio-assembly (reprinted with permission from [89]. Copyright 2011 Royal Society of Chemistry); (**d**,**e**) Amorphous mesoporous FeP particles prepared by a cost-effective electrochemical method without a surfactant (reprinted with permission from [55]. Copyright 2012 Elsevier); (**f**) Hexagonal mesostructured oxovanadium phosphates, ICMUV-2, synthesized by Surfactant templating (CTAB)-directing assembly (reprinted with permission from [90]. Copyright 1999 American Chemical Society); (**g**) Lamellar (VO)_2_P_2_O_7_ crystals obtained by pyrolysis of ICMUV-2 under N_2_ ambience at 973 K (reprinted with permission from [90]. Copyright 1999 American Chemical Society); (**h**) Lamellar- and (**i**) hexagonal-mesostructured VP materials synthesized by assembling exfoliated VOPO_4_ sheets using CTAB as the cationic surfactants (reprinted with permission from [92]. Copyright 2005 Elsevier); (**j**) Spherical NiP nanoparticles with mesopores synthesized by a hydrothermal procedure (reprinted with permission from [95]. Copyright 1993 Elsevier); (**k**) NiPO-1 and (**l**) NiPO-2, with nanotubular structures, derived from sol-gel method (reprinted with permission from [96]. Copyright 2008 Royal Society of Chemistry); (**m**) HPNP-1 synthesized via a surfactant-free hydrothermal method (reprinted with permission from [10]. Copyright 2012 Elsevier).

Transition metal phosphates have been extensively explored for their unique redox properties in heterogeneous catalysis. Iron and vanadium phosphates are undoubtedly the most prominent ones in this regard. In this section, the fabrication of mesostructured iron, vanadium and nickel phosphates will be discussed, as well as the applications, mainly in catalysis. The synthesis conditions, structures and morphologies and applications of respective component are summarized in Figure 5 and Table 3.

Iron phosphate (FeP) has been widely explored as a main component of a solid catalyst in a number of selective oxidation reactions, e.g., ammoxidation of 2-methyl pyrazine [83], oxidative dehydrogenation of isobutyric acid [2], partial oxidation and oxidative bromination of methane reactions [3,4]. The mesoporous FeP with ordered mesostructure (Figure 5a) was first reported by Guo* et al.*, prepared with the HF assembly method [84]. It is emphasized that layer structures rather than hexagonal mesoporous FeP would be obtained without the mediation of fluorate ions. Later, Pillai and Sahle-Demessie [85] found that the as-synthesized mesoporous FeP was a highly active and recyclable heterogeneous catalyst for the selective synthesis of nopol by Prins condensation of *β*-pinene and paraformaldehyde in acetonitrile. After five cycles, no apparent drop in activity was observed, with 100% selectivity to nopol. Nanotubular and mesoporous FeP (Figure 5b) with a specific surface area of 232 m^2^/g and a bimodal distribution of pore sizes were also synthesized in a modified fluoride route with the aid of sodium dodecyl sulfate (SDS) as a template [86]. This novel tubular material can benefit the diffusion of reactive molecules, thus, it showed better catalytic performance for direct hydroxylation of benzene with hydrogen peroxide compared with conventional mesoporous counterpart [86].

On the other hand, iron phosphate is well known as a cathode electrode materials for the low cost, environmentally friendly and high theoretical specific capacity in Li-ion cells [5]. Hexagonally ordered mesoporous FeP materials were also successfully synthesized by using generation 4.0 NH_2_–terminated high generation poly(amido amine) dendrimer (G4-NH_2_) as a single molecular template [87]. Yang* et al.* have synthesized mesoporous FePO_4_ cathode via a surfactant (P123) self-assembly method, which enhanced the lithium ion intercalation/deintercalation kinetics [88]. More recently, a M13 virus-based bio-assembly of amorphous FeP nanowires (Figure 5c) with diameters of 10~20 nm was proposed by Lee and Belcher [89]. By implementing heterostructures with silver atoms, the overall electronic conductivity of the entire composite was greatly enhanced, thereby improving the battery performances [89]. It is obvious that common to all the above mentioned preparative routes is the involvement of a surfactant or a template agent. Using a cost-effective electrochemical method, amorphous mesoporous FeP materials (Figure 5d,e) with particle size ranging from 20 to 80 nm, a surface area of 65.2 m^2^/g and a dominant pore diameter of 23.6 nm have been successfully synthesized without a surfactant [55]. The as-obtained materials were further used to prepare LiFePO_4_/C cathode electrode, exhibiting excellent cycling performances [55].

Vanadium phosphates (VP) represent perhaps the most well studied heterogeneous catalyst and the sole example of a commercialized material for the catalytic oxidation of an alkane since their discovery as an effective catalyst in 1966 [1]. Worldwide, they are used commercially for the production of maleic anhydride and have been extensively studied. A linear relationship has been found between specific butane conversion and surface area of VP catalysts [1]. This highlights the importance to prepare VP catalysts with high surface area. Accordingly, one relatively straightforward approach is to fabricate mesoporous VP materials with high porosity and enhanced surface area. Hexagonal mesostructured mixed-valence oxovanadium phosphates [CTA]*_x_*VOPO_4_·*z*H_2_O (Figure 5f), abbreviated as ICMUV-2, have been synthesized through a S^+^I^−^ cooperative mechanism using CTAB as a template [90]. Pyrolysis of ICMUV-2 at 973 K under flowing N_2_ atmosphere can lead to the formation of (VO)_2_P_2_O_7_ phase, the presumed active phase in selective oxidation of butane. Particles of (VO)_2_P_2_O_7_ consist of a mixture of large lamellar crystals, displaying in some cases hexagonal geometry, and small prismatic crystals in the range of 3–8 µm (Figure 5g). Using a hydrothermal synthesis in the presence of C_16_TMA, hexagonal-, cubic- and lamellar-mesostructured VP materials can be synthesized by tuning the pH value in the ranges of 2.63–2.95, 3.00–3.36 and 3.45–4.45, respectively [91]. Hexagonal- and lamellar-mesostructured VP materials (Figure 5h,i) were also synthesized by assembling exfoliated VOPO_4_ sheets using C*_n_*TAB (*n* = 12, 14, 16, 18) as the cationic surfactants by Kamiya and coworkers [92]. However, it seems very difficult to remove the surfactants completely even after thermal heating at 973 K, above which these mesostructures might collapse [92]. Ion exchange with metal cations might be a promising method for template removal, without regard to the influence of heteroatoms on the catalytic performance.

The synthesis of mesoporous nickel phosphates (NiP) is much more difficult, compared with the preparation of mesostructured iron- and vanadium-phosphates. On one hand, NiO_6 _and PO_4_ differ in bond lengths and angles [93]; on the other hand, nickel and phosphorus species are more apt to precipitation [94]. Kandori* et al.* [95] first synthesized a uniform spherical NiP nanopartical with high mesoporosity (Figure 5j) and a surface area of 130 m^2^/g by using a hydrothermal procedure in the presence of a surfactant SDS. Two types of mesoporous NiP, namely NiPO-1 and NiPO-2, with nanotubular structures (Figure 5k,l) were developed in the presence of CTAB and different bases by a sol-gel method [96].

**Figure 6:**
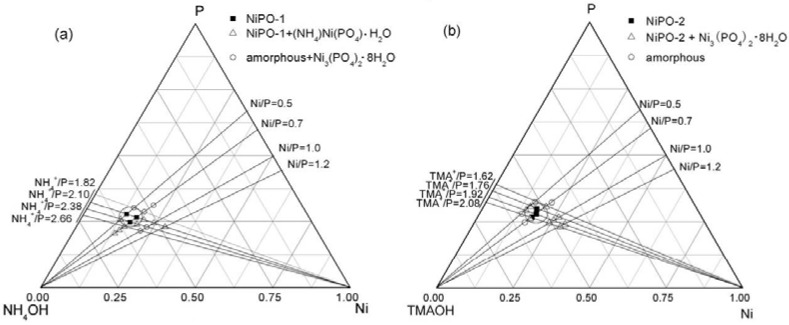
Ternary phase diagrams of the structures obtained in the synthesis of (**a**) NiPO-1; (**b**) NiPO-2 (reprinted with permission from [96]. Copyright 2008 Royal Society of Chemistry).

The synthesis was based on a self-assembly between the cationic surfactant and two precursors (phosphorous and nickel species) in a particular pH range. The phase and textural characteristics are largely dependent on the type of bases and the ratios of P/Ni/base, as shown in the ternary phase diagrams (Figure 6). Both materials possess a relatively high surface area (205~292 m^2^/g). High activity (>50%) and high selectivity to epoxide (95.6% and 99.0%) were achieved in the epoxidation of cyclododecene with H_2_O_2_ as an oxidant [96]. By using an organophosphorus precursor hexamethylenediamine-*N*,*N*,*N'*,*N'*-tetrakis-(methylphosphonic acid) (HDTMP), Dutta* et al.* [10] synthesized a new crystalline porous organic-inorganic hybrid nickel phosphonate material (HPNP-1) in the absence of any template agents via a hydrothermal method. This material possesses a BET surface area of 241 m^2^/g, and the SEM image (Figure 5m) showed that spherical particles in the size range from 25 to 30 nm were uniformly distributed throughout the specimen. Beside high affinity for the adsorption of metal cations, this material showed excellent activity and selectivity in liquid phase reduction of nitrobenzenes to the respective anilines in the presence of NaBH_4_ [10].

**Table 3:** Summary on the synthesis, structural property and application of mesostructured iron, vanadium and nickel phosphates.

Materials	Synthesis conditions	Physical properties	Applications	Reference
FeP	Electrochemical	Nanoparticals, 20–80 nm, S_BET_* ca.* 65 m^2^/g, dominant pore size 23.6 nm	LiFePO_4_/C, cathode materials	[55]
	Solution precipitation, HF, sodium dodecyl sulfate (SDS)	Ordered mesopores, S_BET_ *ca.* 254 m^2^/g, average pore size 2.6 nm	Prins condensation of *β*-pinene and paraformaldehyde	[84,85]
	Solvothermal, SDS-templating assembly	Mesoporous nanotubes, 50~400 nm (diameter), lengths of several microns; S_BET_ 232 m^2^/g	Direct hydroxylation of benzene	[86]
	G4-NH_2_-terminated PAMAM dendrimer, single template assembly	Hexagonal ordering structures	Untested	[87]
VP	Thermal treatment of vanadyl *n*-butylphosphate, 525–705 K	Highly porous, S_BET_* ca.* 225 m^2^/g, mesopore diameter *ca.* 4.4 nm	Precursors of the (VO)_2_P_2_O_7_ catalyst	[59]
	CTAB-templating and/or hydrothermal post-treatment	Hexagonal structures	The same as above	[90]
	Hydrothermal, C_16_TMA(OH,Cl), 473 K, 48 h	Hexagonal-, cubic-, and lamellar-mesostructures	The same as above	[91]
	Surfactant templating (CTAB) of exfoliated VOPO_4_ sheets	Hexagonal- and lamellar-mesostructures	The same as above	[92]
NiP	Hydrothermal, template-free, HDTMP, 443 K, 36 h	Crystalline porous organic–inorganic hybrid, S_BET_ 241 m^2^/g	Adsorption of heavy metal cations like Cr^3+^, Pb^2+^, Hg^2+^ and Cd^2+^; Nitrobenzenes reduction to the respective anilines	[10]
	Hydrothermal, NiSO_4_ + SDS + NaH_2_PO_4_, 353 K	Spherical particles (*ca.* 245 nm) with high porosity, S_BET_ 130 m^2^/g	Selective adsorption of H_2_O	[95]
	Sol-gel, CTAB, aging at 373 K, 24 h	Nanotubular structures, 200~400 nm (length) × 4~5 nm (diameter); S_BET_ 205–292 m^2^/g	Epoxidation of cyclododecene with H_2_O_2_ as an oxidant	[96]

### 3.3. Other Mesostructured Transition Metal Phosphates

**Figure 7:**
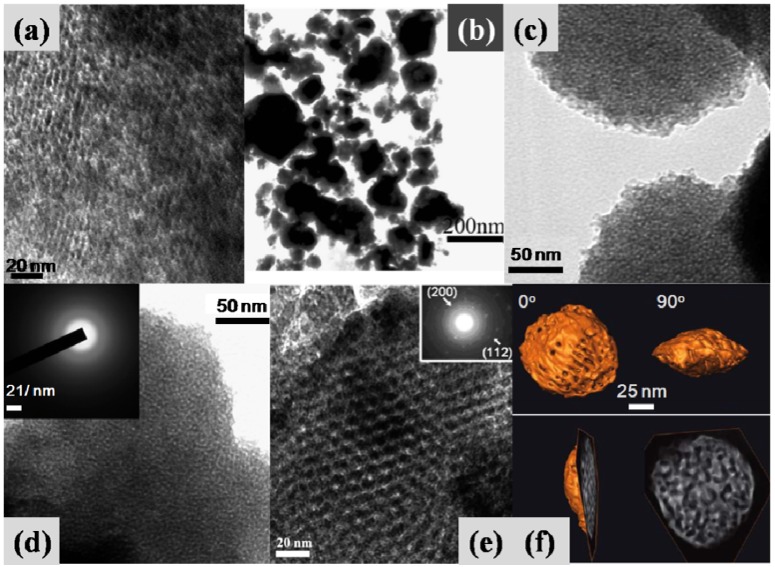
TEM/AFM images of mesoporous transition metal phosphates materials. (**a**) Less ordered mesoporous chromium phosphate (CrP) prepared by ball milling (reprinted with permission from [99]. Copyright 2012 Royal Society of Chemistry); (**b**) Zinc phosphate (ZnP) nanoparticles synthesized by bio-assembly (reprinted with permission from [103]. Copyright 2009 Elsevier); (**c**) Niobium oxophosphates (NbP) with wormhole-like morphologies prepared by tetradecyltrimethylammonium bromide (TTBr)-assisted precipitation (reprinted with permission from [100]. Copyright 2009 Elsevier); (**d**) Amorphous tantalum phosphate (TaP) synthesized via a sequential precipitation-hydrothermal treating technique (reprinted with permission from [104]. Copyright 2010 Elsevier); (**e**) Cubic ordered mesoporous yttrium phosphates (YP)-derived by nanocasting with KIT-6 as a hard template (reprinted with permission from [12]. Copyright 2009 Royal Society of Chemistry); (**f**) Mesoporous YP with lenticular morphology, prepared by microwave-assisted precipitation (reprinted with permission from [11]. Copyright 2012 American Chemical Society).

Due to the unique properties of TMP materials, the synthesis methods of other mesoporous transition metal phosphates with novel structural and morphological features are occasionally reported. Correspondingly, the synthesis conditions, structures and morphologies and applications of these less studied components are summarized in Figure 7 and Table 4.

**Table 4:** Summary on the synthesis, structural property and application of other mesostructured transition metal phosphates.

Materials	Synthesis conditions	Physical properties	Applications	Reference
CrP	Sol-gel combined with programmed hydrothermal treating, TTBr	Ink-bottle pores, S_BET_ 384 m^2^/g, average pore size 3.6 nm	Untested	[98]
	Solid-state reaction at 373 K, CTAB	Banger-like pores, S_BET_ 250 m^2^/g, average pore size 3.48 nm	Isopropanol dehydration to propene	[99]
NbP	Hydrothermal, TTBr as template, aging at 403 K, 24 h	Wormhole-like structure, S_BET_ 427 m^2^/g, average pore size 3.35 nm	Potential solid acid catalyst	[100]
	Precipitation combined with hydrothermal treating, CTAB	Wormhole-like structure, S_BET_ 210~290 m^2^/g, average pore size 3.5 nm	Isomerization of xylose to xylulose and subsequent dehydration; dehydration of fructose to 5-hydroxymethylfurfural	[101,102]
ZnP	Chemical precipitation with yeast cells as biotemplates, pH 8~10	Agglomerates of isolated nanoparticles of 10 nm, S_BET_ 146 m^2^/g, average pore size 10 nm	Untested	[103]
TaP	Precipitation combined with hydrothermal treating, TTBr	Wormhole-like structure, S_BET_ 324~359 m^2^/g, average pore size 2.7~3.5 nm	Untested	[104]
YP	Microwave-assisted precipitation	Lenticular nanoparticles with internal porosity and a pore diameter of *ca.* 4.0 nm, S_BET_ 145 m^2^/g	Photoluminescence material	[11]
	Nanocasting route, KIT-6 as hard template	Cubic ordered mesopores, S_BET_ 114 m^2^/g, average pore size 4.3 nm	The same as above	[12]

Mesoporous chromium phosphate (CrP) has been synthesized with the aid of surfactant tetradecyltrimethylammonium bromide (TTBr), using a sequential sol-gel and hydrothermal technique [98]. The as-synthesized material exhibits a relatively high specific surface area of 384 m^2^/g and a narrow pore size distribution centered at 3.6 nm, but no apparent structural patterns can be identified from TEM observations. Ball milling of CrCl_3_·6H_2_O + NaH_2_PO_4_·2H_2_O + CTAB, followed by template removal with calcination in N_2_ at 823 K, can produce mesoporous CrP with banger-like pore geometry (Figure 7a) [99]. The mesoporous CrP possessed a specific surface area as high as 250 m^2^/g. When used in the dehydration of isopropanol to propene, these catalysts exhibited significantly higher isopropanol conversions and propene yields than those synthesized via the solution sol-gel route and the non-mesoporous chromium phosphate [99].

Mesoporous niobium oxophosphates (NbP) with wormhole-like morphologies (Figure 7c) and a high specific surface area of 427 m^2^/g were prepared using aqueous solution of niobium tartrate complex and (NH_4_)_2_HPO_4_ as precursors and TTBr as a template [100]. Both Brønsted and Lewis acid sites existed on the surface of materials, as confirmed by means of pyridine adsorption [100].

Combining the biomineralization technology and crystals assembly on biomacromolecules, He and coworkers [103] reported the synthesis of mesoporous zinc phosphate (ZnP) crystals, with isolated tiny particles of 10 nm in size forming soft agglomerates of varied shapes (Figure 7b). Hopefully, this technique might be expanded to the synthesis of other less ordered mesoporous metal phosphates, but morphology control presents a huge challenge.

Mesoporous tantalum phosphate (TaP) has been synthesized through a sequential precipitation-hydrothermal treating technique using aqueous solution of tantalum tartrate complex and diammonium hydrogen phosphate as precursors of tantalum and phosphorous, respectively, and a cationic surfactant TTBr as a structure directing agent [104]. The obtained mesoporous material showed the amorphous nature with wormhole-like structure (Figure 7d), a high surface area 359 m^2^/g and a narrow pore size in the range of 2.3~10 nm.

Using nanocasting technology, Luo* et al.* [12] successfully synthesized cubic ordered mesoporous yttrium phosphates (YP) (Figure 7e), with a Y(NO_3_)_3_/H_3_PO_4_/HNO_3_ system as a guest unit and KIT-6 as a hard template. After doping with appropriate lanthanide ions (such as Eu, Tb and Ce), cubic ordered mesoporous RE:YPO_4_ composites with tunable intense optical properties (such as orange-red, green and blue emission) can be obtained. Quite lately, mesoporous tetragonal YPO_4_ and RE:YPO_4_ (RE = Eu, Tb, Ce, and Ce + Tb) composites with a lenticular morphology (Figure 7f), narrow size distribution and high surface area were prepared by a simple and rapid homogeneous precipitation procedure assisted by microwave radiation [11]. Such mesoporous RE:YPO_4_ materials, having both the mesoporous properties for the storage of biological actives and photoluminescence properties for the online monitoring of carrier, might be applied as a new generation of drug delivery vehicles in biomedicine [13,14].

## 4. Conclusions and Future Perspectives

With the rapid development of modern fabricating techniques, great success has been achieved in the preparation of a diverse range of mesostructured silica materials, such as spheres, fibers, rods, thin films and hollow nanospheres [105]. Significant progress has been made in the shift to mesoporous non-silicate materials since the first report of M41S [34]. Mesoporous transition metal phosphates, due to their unique properties and potential applications in optical element, proton conducting devices, biomimetic chemistry, separation and catalysis, have been extensively investigated. By far, a large number of mesoporous transition metal phosphates with various morphologies and porous structures can be obtained mainly by a templating route. But, there are still some problems, which confine the further developments. (i) Liquid crystal template theory, well-understood in the synthesis of mesostructured silicate materials, is far unclear in tri-elemental M–P–O systems (M for metals). Self-assembly is much more complex, considering two totally different types of precursors involved in the system, when compared with that in Si–O system. Besides, inorganic metal precursors are commonly used in the surfactant-assisted templating route. The hydrolysis and condensation processes are much faster for metal ions than organic-inorganic hybrid silicates, presenting a big challenge for the controlled assembly of mesoporous transition metal phosphates with certain aimed morphologies. In some cases, precipitation can easily occur when both metal and phosphorus precursors are added in a solution [12,93]. For these reasons, there has been very few reports concerning the control of specific morphologies in mesostructured transition metal phosphate materials. (ii) For non-silicate materials, conservation of a mesoporous structure after removal of structure-directing agents still remains a challenge. In a typical sol-gel synthesis, organic templates used in the preparation of mesostructured transition metal phosphates are usually removed by high-temperature calcination, acid-ethanol extraction or both [65]. Ordered structures of the metal phosphate-surfactant composites would be easily destroyed after thermal treatment in a high temperature region, resulting in a less ordered structure. Acid-ethanol extraction is a relatively mild method. However, this usually leads to the incomplete removal of organic species, and the residuals might restrict the applications of these mesoporous materials in some particular fields due to their harmful effects. Exploring other strategies to the synthesis of well-ordered mesoporous transition metal phosphate materials still remains an interesting topic. (iii) The fabrication of mesostructured transition metal phosphate materials is mainly confined to zirconium and titanium phosphates, while the other metal phosphates are rarely reported. This reflects the facile applications of mesoporous ZrP and TiP in large areas, as well as the relatively simple preparation, at least in part. Thus, great efforts should be devoted to develop novel strategies for synthesizing other mesoporous transition metal phosphates, a prerequisite of further exploring their potential applications. It should be kept in mind that post-modification of these mesoporous metal phosphonate materials with organic and/or inorganic molecules might be a simple and effective route for tailoring the surface properties, taking advantage of the versatile functional groups of phosphates (PO_4_^3−^, HPO_4_^2−^ and H_2_PO_4_^−^) or P–OH.
